# Transvaginal radiofrequency ablation for uterine myomas: A preliminary ecuadorian study

**DOI:** 10.1016/j.eurox.2025.100443

**Published:** 2026-01-02

**Authors:** Hernan Sabay, Maritza Freire, Belen Tite, Eduardo Pilatuna, Paola Solis- Pazmino

**Affiliations:** a Instituto de Radiofrecuencia (ITECC), Quito, Ecuador; b Pontificia Universidad Catolica del Ecuador (PUCE), Posgrado de Ginecologia y Obstetricia, Quito, Ecuador; c Universidad San Francisco de Quito, School of Medicine, Quito, Ecuador; d The Surgery Group of Los Angeles, 8635 W 3rd St #880, Los Angeles, CA 90048, USA; e Division of General Surgery, Santa Casa de Misericordia, Porto Alegre, Brazil

**Keywords:** Transvaginal radiofrequency ablation, Uterine myoma, Volume reduction, Complications, Minimally invasive procedure

## Abstract

**Background:**

Transvaginal radiofrequency ablation (TVRFA) is a minimally invasive, incisionless, outpatient procedure. This study evaluates the feasibility, outcomes, and complications of TVRFA in women with symptomatic uterine myomas (UMs).

**Methods:**

This preliminary single-center, retrospective study included 79 women treated with TVRFA between December 2021 and April 2024 in Quito, Ecuador. Women with 1–4 UMs of any size or type were eligible. Preoperative and follow-up assessments included transvaginal ultrasound (TVUS) for both uterine and myoma volume. Outcomes (uterine and myoma volume + complications) were assessed at 45 days and 6 months. Complications were classified by Clavien–Dindo. Statistical analyses used paired tests for baseline vs. follow-up comparisons, with missing data excluded from each analysis.

**Results:**

The median baseline uterine volume was 160.0 mL. At 6 months, this decreased to 91.0 mL (−43.1 %, p < 0.001). The median baseline myoma volume was 22.5 mL, which decreased to 7.7 mL at 45 days (−55.1 %) and 3.7 mL at 6 months (−81.5 %, p < 0.001). Follow-up was completed by 68/79 patients (86.1 %) at 45 days and 59/79 (74.7 %) at 6 months. Minor complications occurred in 12.7 %, most commonly skin burns at the electrode site. Two patients (2.5 %) experienced intestinal perforation requiring surgery.

**Conclusion:**

TVRFA significantly reduced both uterine and myoma volumes, supporting its role as a minimally invasive alternative for fibroid management. However, the observed complication rate, particularly intestinal perforation, requires critical attention to operator training and procedural refinements. Prospective studies with standardized symptom measures are warranted.

## 1 Introduction

The prevalence of uterine myomas (UMs) ranges from 20 % to 40 % between puberty and menopause [1]. UMs, also known as fibroids, are non-cancerous tumors that develop within or around the uterus, and their location often determines the fibroid type and associated symptoms, including heavy menstrual bleeding, pelvic pain, and reproductive complications [2], [3].

Treatment options for symptomatic UMs vary depending on factors such as the size, location, and number of UMs, as well as the patient's symptoms, age, and desire for future fertility [4]. A wide range of management options is available, including medical therapy, non-excisional procedures, and surgical interventions [5].

Radiofrequency ablation (RFA) is a minimally invasive treatment option for UMs that delivers controlled radiofrequency energy (heat) to fibroid tissue, causing coagulative necrosis that is reabsorbed over time, leading to shrinkage [6]. RFA offers benefits such as shorter recovery times, preservation of the uterus, and significant symptom relief, making it an attractive alternative to more invasive procedures like hysterectomy [6]. RFA works by generating heat, and destroying UM tissue and can be delivered laparoscopically, transvaginally, or transcervically [7], [8].

A systematic review of RFA for UMs has shown that most studies performed RFA by a laparoscopic approach [9], [10]. A minority of studies evaluated transvaginal RFA (TVRFA) procedures, although this approach was associated with a shorter procedure time and length of stay compared with laparoscopic RFA [11]. Currently, there is limited evidence on the TVRFA approach, with only a few studies reporting outcomes and complications [12], [13]. This study aimed to evaluate the feasibility, outcomes, and complications of TVRFA among patients with symptomatic UMs.

## 2 Methods

### 2.1 Study design and participants

This preliminary retrospective, single-center study included 79 women with symptomatic UMs who underwent TVRFA at a private hospital in Quito, Ecuador, between December 2021 and April 2024. All procedures were performed by two experienced gynecologic surgeons. Although the present manuscript focuses on this initial cohort, patient recruitment and follow-up are ongoing as part of a larger study, with a total of 392 women currently enrolled.

Inclusion criteria: women ≥ 18 years with one to four myomas of any size or FIGO type or symptomatic uterine myomas.

Exclusion criteria: > 4 myomas, malignancy or precancerous conditions, pelvic inflammatory disease (PID), coagulopathy, or pregnancy.

The study was approved by the Ethics Committee of the Universidad San Francisco de Quito (E01-EX0232–2024-23153M-CEISH-USFQ) and conducted according to the ethical principles of the Declaration of Helsinki (seventh revision). The manuscript followed the STROBE guidelines for observational studies.

### 2.2 Ablation procedure

Before treatment, all patients had complete blood count, thyroid function tests, coagulation tests, and transvaginal ultrasound (TVUS) evaluation. A TVUS evaluated the number, volume, and location of the myomas.

RFA procedures were performed on patients under general anesthesia. All procedures were in an outpatient setting. Patients were positioned in the lithotomy position, with a dispersive electrode pad on the anterior thigh. The RFA system includes the VIVA RF System Generator (STARmed,) and a coagulation electrode with a coolant system to maintain impedance. The generator operates at 480 kHz with up to 200 W of power and was set to a maximum of 100 W during the procedure. It displays the electrode tip temperature, tissue impedance, and ablation time. Using transvaginal ultrasound guidance, a 35 cm long 18-gauge needle electrode was inserted via a needle guide on the ultrasound probe. The procedure involved finding the safest and shortest path to the target myoma, which could involve passing through the posterior fornix, myometrium, or other myomas. The electrode's exposed tip was precisely positioned at the center of the myoma to ensure accurate targeting for treatment. The ablation points were mapped along a line inside the myoma, starting from the distal end and moving proximally, with overlapping areas. The core of the target myoma was ablated once the echo-enhanced region covered 80 %–90 % of the myoma's cross-sectional area on real-time ultrasound.

Patients were observed for 2–3 h after the procedure and discharged if they were alert, able to consume fluids and food, and not experiencing excessive pain. They were prescribed nonsteroidal anti-inflammatory drugs and antibiotics for 5 days to prevent infection in the necrotic myoma tissue [14].

### 2.3 Data collection and variables

We used the medical records to collect the following variables: 1) Demographic details (age, education, residence, diagnosis age, ethnicity); 2) family history of gynecological cancer; 3) comorbidities; 4) symptoms related to UMs (abnormal uterine bleeding, dyspareunia, dysmenorrhea); 5) pre-operative blood test; 6) pelvic ultrasound characteristics; 7) RFA characteristics (power, time, and energy); 8) Complications.

### 2.4 Data management

UMs volume was calculated via transvaginal ultrasound using the formula Volume = 0.5233 × L × W × H, and the volume reduction at forty-five days and six months was calculated as (initial volume – final volume) × 100 / initial volume.

Complications were deﬁned according to the Clavien–Dindo classiﬁcation [15].

We employed the International Federation of Gynecology and Obstetrics (FIGO) classification system to describe the type of myoma as follows: transmural (FIGO 2–5), intramural (FIGO 3–4), submucosal (FIGO 1–2), and subserosal (FIGO 5–6) [16].

Abnormal uterine bleeding was defined as irregularities in the menstrual cycle involving frequency, regularity, duration, and volume of flow outside of pregnancy [17]. Dysmenorrhea was defined as pain during the menstrual cycle [18]. Dyspareunia was defined by genital pain that can be experienced before, during, or after intercourse [19].

### 2.5 Follow-up evaluation

Patients were followed up at 45 days and 6 months after index RFA, per the routine clinic practice. During each appointment, repeat TVUS and laboratory tests were performed. The volume reduction (VR) was determined using a calculator [20].

### 2.6 Outcome measures

The primary outcome included uterine volume reduction and treated myoma volume reduction at 45 days and 6 months. The secondary outcome was complication rates.

### 2.7 Statistical analysis

Continuous variables were expressed as mean and standard deviation (SD) when normally distributed or otherwise as the median and interquartile range (IQR). The Student *t*-test and the Mann-Whitney *U* test were used as appropriate to analyze continuous variables. Categorical data were expressed in the form of numbers and percentages and were analyzed using the Fisher exact test or Chi-Square test. UMs volume at baseline was compared with that at the six-month follow-up using the Wilcoxon test. A p-value of.05 was considered to indicate significance. Statistical analysis was performed by using the SPSS program (version 24.0)

## 3 Results

### 3.1 Demographic characteristics

Seventy-nine women were included in the study (Table 1). The median age was 41 years. A third of patients were overweight (n = 17; 37.8 %). Reported baseline symptoms included abnormal uterine bleeding in 31 patients (52.6 %), dysmenorrhea in 23 (38.9 %), and dyspareunia in 5 (8.5 %). Anemia was present in 10 patients (12.7 %), and hypothyroidism in 7 patients (8.9 %).

**Table 1:** Baseline characteristics (n = 79).

**Variable**	
**Age at diagnosis**	41.0 [37.0–45.0]
**Residence**	
Coast	10 (10.3)
Highland	64 (83.5)
Amazonia	3 (4.1)
Galapagos	1 (0.7)
Other than Equator	1 (1.4)
**Employment**	
Domestic chores	18 (19.8)
Public employee	61 (80.2)
**Education level**	
High school	20 (12.5)
University	59 (87.5)
**BMI**	
Normal (18.5–24.9 kg/m^2^)	21 (46.7)
Overweight (25.0–29.9 kg/m^2^)	17 (37.8)
Obesity class 1 (30.0–34.9 kg/m^2^)	7 (15.5)
**Comorbidities**	
Anemia	10 (12.7)
Hypothyroidism	7 (8.9)
Hypertension	2 (2.5)
PCOS	2 (2.5)
**Symptoms**	
Dysmenorrhea	23 (38.9)
Abnormal uterine bleeding	31 (52.6)
Dyspareunia	5 (8.5)
**Hemoglobin**	12.9 [12.0–4.1]
**Hematocrit**	37.6 [35.2–38.4]
**Fibroid type**	
Transmural (FIGO 2–5)	4 (5.3)
Intramural (FIGO 3–4)	54 (72.0)
Submucosal (FIGO 1–2)	9 (12.0)
Subserosa (FIGO 5–6)	8 (10.7)

Continuous variables are expressed as median [interquartile range]

Categorical variables are presented as proportions (%)

**Abbreviation:** BMI: body mass index

### 3.2 Pre-ablation assessment

Most myomas were intramural (n = 64; 72 %), followed by submucosal (n = 9; 12 %), subserosal (n = 8; 10.7 %), and transmural (n = 4; 5.3 %). The median number of myomas per patient was 3, with a median of 2 treated per procedure.

### 3.3 Procedure characteristics

All procedures were performed under general anesthesia (Table 2). The median procedure time was 7.4 min, the power setting was120 W and the energy delivery was 28.9 J.

**Table 2:** Treatment parameters of radiofrequency ablation (n = 79).

**Variable**	
**Anesthesia**	
General anesthesia	79 (100)
**RFA machine**	
Energy median (J) (n = 54)	28.87 [0.47–45.8]
Time median (min) (n = 89)	7’ 40’’ [0.44–60]
Power median (W)	120 [39.9 – 738.5]
**Number of sessions**	
Single	165 (100)

Continuous variables are expressed as median [interquartile range]

Categorical variables are presented as proportions (%)

Abbreviations: J: joules; W: watts

### 3.4 Volume reduction (VR)

#### 3.4.1 Uterine volume

At baseline, the median uterine volume was 160.0 mL. At 45 days, this decreased to 114.5 mL, representing a 33.2 % reduction (p < 0.001). At six months, the uterine volume further decreased to 91.0 mL, corresponding to a 43.1 % reduction (p < 0.001).

#### 3.4.2 Myomas

**Fig. 1:**
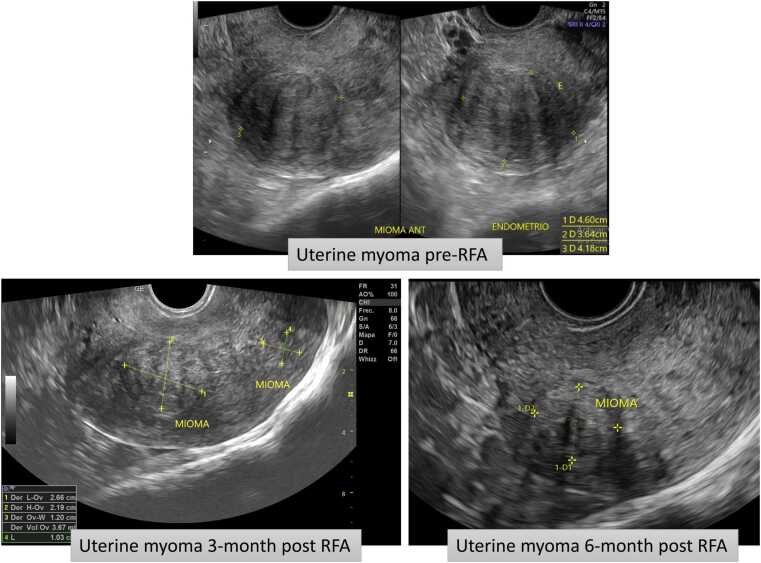
Volume reduction of the uterine myoma.

The median baseline volume of treated myomas was 22.5 mL. At 45 days, this decreased to 7.7 mL, corresponding to a 55.1 % reduction (p < 0.001). At six months, the median volume was 3.7 mL, reflecting an 81.5 % reduction (p < 0.001) (Table 3a and b, Fig. 1). At 45-day follow-up, 68/79 patients (86.1 %) had complete ultrasound data; at 6 months, 59/79 (74.7 %) completed follow-up.

**Table 3:** Volume reduction at 45 days and 6 months follow- up.

**Variables**	**Baseline**	**45 days**	**P value**	**6 months**	**P value**
**Uterus volume (mm**^**2**^**)**	160.0 [115.3–254.0] **(n = 72)**	114.5 [67.3–153.8] **(n = 72)**	< .001	114.5 [80.5–129.5] **(n = 32)**	< .001
**Median uterus volume reduction, %**		33.2 [20.8–49.3]		43.1 [27.0–57.0]	
**Myoma volume (mm**^**2**^**)**	22.5 [5.7–72.9] **(n = 62)**	7.7 [2.3–23.4] **(n = 62)**	< .001	3.7 [0.7–11.6] **(n = 24)**	< .001
**Myoma volume reduction, %**		55.1 [37.9–78.1]		81.5 [59.5–94.1]	

Data are Median [Interquartile Range]

#### 3.4.3 Complications

Overall, 16.5 % of patients experienced complications (Table 4). Minor complications included device-related first-degree skin burns at the dispersive electrode site in 10 patients (12.7 %, Clavien–Dindo I) and one case of pelvic inflammatory disease requiring intravenous antibiotics (1.3 %, Clavien–Dindo II). Major complications occurred in two patients (2.5 %), who developed intestinal perforation, both requiring ileocolectomy and primary anastomosis (Clavien–Dindo IIIb).

**Table 4:** Clavien- Dindo classification.

**Clavien- Dindo**	**N (%)**	**Type of complication**	**Time to onset**	**Treatment**
I	10 (12.65)	first-degree skin burn	Immediate	cool water, pain medication
II	1 (1.27)	pelvic inflammatory disease	Immediate	IV Antibiotics
IIIb	2 (2.53)	intestinal perforation	Immediate	laparotomy (ileocolectomy and primary anastomosis).

## 4 Discussion

This study demonstrates that transvaginal radiofrequency ablation (TVRFA) leads to substantial reductions in both uterine and myoma volumes. Treated myomas decreased by 55.1 % at 45 days and 81.5 % at 6 months, while uterine volume was reduced by 43.1 % at 6 months.

Several studies have supported the efficacy and safety of radiofrequency ablation (RFA) for treating UMs. Most studies evaluated the laparoscopic approach. In a prospective study of 114 patients, Robles et al [20]. reported a 48.2 mL volume decrease at 12-month follow-up. A retrospective multicenter study by Galen et al [21]. including 206 women showed that the mean VR decreased from 204.4 mL to 151.4 mL at 12 months.

Although there is substantial evidence supporting RFA by the laparoscopic approach, studies on the transvaginal approach are limited. Recently, a systematic review compared RFA by laparoscopic, transvaginal, and transcervical approaches. They did not find a difference in the VR, reporting that it was 4 % greater with the transcervical and 10 % greater with the transvaginal RFA approach compared with the laparoscopic approach at 12 months. In contrast to the non-significant difference in VR, TVRFA was associated with shorter procedure time (p ≤ 0.002) and length of stay (p < 0.001) [9].

Our study focused on the transvaginal approach and demonstrated a significant VR in UMs in the follow-up. These findings are largely consistent with prior studies, such as those by Yin et al [22], in a retrospective study of 1216 patients treated with TVRFA, who reported a reduction of 39.8–88.3 % over 24 months, and Wu et al [23], in a prospective study of 62 patients, who observed a mean VR of 78 % at 12 months. Notably, the 81.5 % reduction observed at 6 months in the current study is comparable to the 12-month outcomes reported by Wu et al [23], suggesting that significant UM shrinkage may occur earlier than previously documented. The slight variations in early-stage VR, such as the 55.1 % at 45 days, could be due to differences in patient populations, UM characteristics, or RFA techniques.

Regarding the inclusion of fibroids of any size, it is important to note that fibroids smaller than 6 cm are generally considered optimal for radiofrequency ablation. While Rey et al [12]. limited their analysis to intramural fibroids smaller than 5 cm and FIGO types 2–3, and Santalla et al [13]. included fibroids larger than 7 cm (FIGO 0–4), our study encompassed all FIGO types and any fibroid size. The reason was that the primary treatment goal for most patients was symptom relief rather than fertility preservation. Many women sought to alleviate pain, bladder discomfort, or gastrointestinal symptoms associated with mass effect. Even in larger fibroids, radiofrequency ablation can effectively reduce volume and consequently improve these symptoms, regardless of initial size. This decision reflects real-world clinical practice, where many fibroids are not accessible through conventional surgical approaches such as laparoscopy or open myomectomy. In these cases, a percutaneous or transvaginal radiofrequency ablation approach offers a minimally invasive alternative. Rather than performing an extensive surgical resection, reducing the volume of FIGO type 5, 6, or even fixed pedunculated type 7 fibroids can substantially improve patient comfort while avoiding the risks associated with open surgery.

Complications following TVRFA for uterine myomas are generally low, with both minor and major adverse events being relatively uncommon. Most of the studies [14], [24], [25], [26] reported minor complications, such as pain, vaginal discharge, first-degree skin burns or localized discomfort, in 2–8 % of cases [27]. Our study reported that 12.7 % of patients experienced device-related first-degree skin burns, which is within the expected range but highlights the need for improved thermal management protocols during the procedure.

TVRFA major complications were reported by few studies [12], in a retrospective study of 205 women, reported two (0.98 %) cases with type III-b complications (Clavien–Dindo classification) treated with hysteroscopy at 30 and 45 days, respectively, to remove an intracavitary free myoma [13], in a retrospective study of 115 patients treated with TVRFA, reported one case of intestinal perforation requiring surgery and intestinal resection. In our study, two patients (2.5 %) experienced intestinal perforation, treated with laparotomy (ileocolectomy and primary anastomosis). We acknowledge that the reported complication rates are higher than those reported in studies using newer commercial transcervical radiofrequency ablation devices like Sonata System (Gynesonics, Redwood City, CA) or Acessa (Hologic, Inc., Marlborough, MA), approved by the Food and Drug Administration (FDA) [28]. In addition, other thermal ablation like percutaneous microwave ablation (PMWA) has demonstrated minimizing ablation time and avoiding harm to adjacent organs and the endometrium [29].

To potentially decrease the risk of complications, we recommend avoiding multiple insertions of the needle electrode into the myoma. We believe that a single insertion, guided by transvaginal ultrasound, can help minimize the risk of post-procedural intraperitoneal adhesions [26]. Future research should aim at refining procedural techniques, developing advanced thermal protection methods, and conducting larger, multicenter trials to validate complication rates across diverse populations and settings. This will be critical for reducing device-related minor complications and minimizing the risk of rare, but significant major adverse events.

Our study has certain limitations. Firstly, the relatively small sample size limits the statistical power of our findings and may not fully capture the variability in outcomes across different fibroid types and sizes. Second, our follow-up period of 6 months is shorter than that of other studies reporting outcomes up to 24 months. Extended follow-up is ongoing to evaluate the durability of fibroid volume reduction and symptom relief. Also, the loss of data in the follow-up limited the strength of our conclusions, and the short follow-up period prevented us from assessing the long-term effects of RFA. Additionally, the single institution setting and the performance of the procedures by only two providers limit the generalizability of the results. In addition, symptom improvement like dyspareunia, dysmenorrhea, and abnormal uterine bleeding is a critical measure of treatment success. Unfortunately, due to the retrospective nature of this study, consistent and standardized data on symptom outcomes were not available for all patients.

Nevertheless, this study significantly contributes to the literature by demonstrating the consistent volume reduction of UMs over time. To our knowledge, this is one of the first studies to evaluate the vaginal approach for radiofrequency ablation in an Ecuadorian population. Our findings suggest that the technique holds promise and could benefit patients, warranting further investigation with larger sample sizes and longer follow-ups to validate our preliminary results.

## 5 Conclusion

TVRFA for uterine myomas demonstrates significant reductions in both uterine and myoma volumes. These findings support TVRFA as a viable, outpatient, minimally invasive alternative for the management of symptomatic UMs, particularly valuable in settings where access to newer or more advanced technologies is limited. Further research is warranted to refine the technique, minimize complications, and explore long-term outcomes, particularly concerning reproductive health.

## CRediT authorship contribution statement

**Solis Pazmino Andrea Paola:** Writing – review & editing, Writing – original draft, Visualization, Validation, Supervision, Software, Resources, Project administration, Methodology, Investigation, Formal analysis, Data curation, Conceptualization. **Hernan Sabay:** Writing – review & editing, Visualization, Validation, Supervision, Resources, Project administration, Funding acquisition, Data curation, Conceptualization. **Belen Tite:** Writing – review & editing, Methodology, Investigation, Funding acquisition. **Maritza Freire:** Writing – review & editing, Validation, Resources, Investigation. **Eduardo Pilatuna:** Writing – review & editing, Software, Project administration, Methodology, Investigation, Data curation.

## Ethical approval

This study was performed in line with the principles of the Declaration of Helsinki. The Ethics Committee of Universidad San Francisco de Quito approved.

## Consent to participate

Informed consent was obtained from all individual participants included in the study.

## Funding

The authors declare that no funds, grants, or other support were received during the preparation of this manuscript.

## Declaration of Competing Interest

The authors declare that they have no known competing financial interests or personal relationships that could have appeared to influence the work reported in this paper.
